# Risk factors of new vertebral compression fracture after percutaneous vertebroplasty or percutaneous kyphoplasty

**DOI:** 10.3389/fendo.2022.964578

**Published:** 2022-08-31

**Authors:** Yuanpei Cheng, Xiaokang Cheng, Han Wu

**Affiliations:** ^1^ Department of Orthopeadics, China-Japan Union Hospital of Jilin University, Jilin, China; ^2^ Department of Orthopaedics, Beijing Tongren Hospital Affiliated to Capital Medical University, Beijing, China

**Keywords:** osteoporotic vertebral compression fracture, vertebral compression fracture, osteoporosis, percutaneous vertebroplasty, percutaneous kyphoplasty, risk factor

## Abstract

**Background:**

New vertebral compression fracture (VCF) may occur in patients who underwent percutaneous vertebroplasty (PVP) or percutaneous kyphoplasty (PKP) for osteoporotic vertebral compression fracture (OVCF). However, the risk factors of new VCF remain controversial. The research aimed to analyze the risk factors of new VCF after PVP or PKP.

**Methods:**

From August 2019 to March 2021, we retrospectively analyzed the patients who underwent PVP or PKP for OVCF at our institution. Age, gender, body mass index (BMI), smoking, drinking, hypertension, diabetes, fracture location, surgical method, Hounsfield unit (HU) value, preoperative degree of anterior vertebral compression (DAVC), bisphosphonates, bone cement volume, bone cement leakage, and cement distribution were collected. The risk factors were obtained by univariate and multivariate analysis of the data.

**Results:**

A total of 247 patients were included in the study. There were 23 patients (9.3%) with new VCF after PVP or PKP. Univariate analysis showed that age (*p* < 0.001), BMI (*p* = 0.002), fracture location (*p* = 0.030), and a low HU value (*p* < 0.001) were significantly associated with new VCF after PVP or PKP. A low HU value was an independent risk factor for new VCF after PVP or PKP obtained by multivariate regression analysis (OR = 0.963; 95% CI, 0.943–0.984, *p* = 0.001).

**Conclusions:**

In this study, a low HU value was an independent risk factor of new VCF after PVP or PKP.

## Introduction

Osteoporotic vertebral compression fracture (OVCF) is a common disease, with an incidence of 0.307% among people over the age of 50 (1). OVCF is likely to result from low-energy trauma and may cause severe back pain and kyphosis, and even increase mortality (2, 3). Surgery is an alternative therapy for patients who have undergone failed conservative treatments such as analgesia, bed rest, and physical support.

Percutaneous vertebroplasty (PVP) was first introduced to treat vertebral angioma by Galiebert in 1987 (4). Nowadays, PVP and percutaneous kyphoplasty (PKP), with safety and efficacy, are widely used in the treatment of patients with OVCF (5–7). However, some studies reported that adverse events such as cement leakage, pulmonary cement embolism, and new vertebral compression fracture (VCF) occurred after PVP or PKP (8–10).

Currently, new VCF in patients who underwent PVP or PKP has aroused widespread concern (11). Several studies reported that some potential risk factors such as age, gender, body mass index (BMI), bone mineral density (BMD), bone cement volume, and bone cement leakage were associated with new VCF (12–14). However, the risk factors for new VCF are still controversial, especially whether PVP or PKP itself increases the risk of new VCF (11, 15, 16). The purpose of the research is to investigate and identify the risk factors of new VCF and provide clinical guidance for spinal surgeons so as to prevent the recurrence of new VCF in patients treated with PVP or PKP for OVCF.

## Materials and methods

### Study subjects

The study retrospectively reviewed the patients who underwent PVP or PKP for OVCF from August 2019 to March 2021 at our institution. The study obtained the support of the Ethics Committee of our institution and informed consent of all patients, and was also in accordance with the Declaration of Helsinki. These patients were divided into new VCF and control groups according to whether they have or do not have new fractures. The inclusion criteria included definite back pain in line with VCF, single-segment fresh VCF in the elderly patient diagnosed by computed tomography (CT) and magnetic resonance imaging (MRI), low-energy trauma, initial treatment with PVP or PKP, and a follow-up time of at least 12 months. The exclusion criteria included initial treatment with PVP or PKP for multi-segment VCFs, neurological symptoms, spinal cord compression, pathological fracture, vertebral burst fractures, posterior column fracture, high-energy trauma, spinal infection, spinal tumor, spinal tuberculosis, incomplete image data, and loss to follow-up.

### Surgical procedure

The patient was placed in the prone position, and the procedure was performed under local anesthesia. The surgical segment was confirmed under fluoroscopic guidance. The entry point was determined on the skin surface. A puncture needle was advanced to the fractured vertebral body by unilateral pedicle with the aid of fluoroscopic guidance. If necessary, a balloon was inserted to restore vertebral height. The prepared bone cement was injected into the fractured vertebral body under fluoroscopy. If bone cement leakage was observed, the operation should be stopped immediately.

### Measures

**Figure 1:**
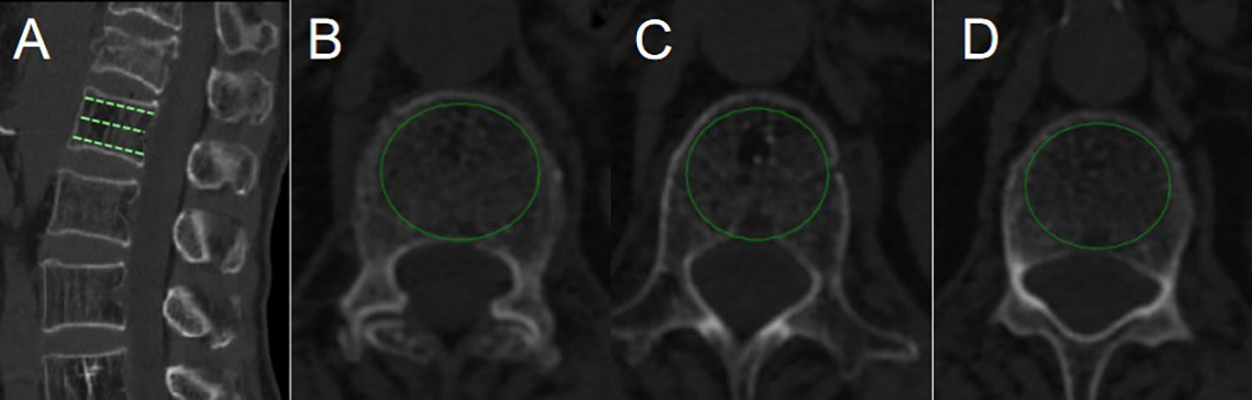
HU value was measured by PACS with the use of an elliptical region of interest function. **(A)** was a reconstructed CT sagittal image, marking the positions of the three slices. Slice **(B)** was chosen just inferior to the superior endplate. Slice **(C)** was taken in the middle of the body. Slice **(D)** was chosen just superior to the inferior endplate.

**Figure 2:**
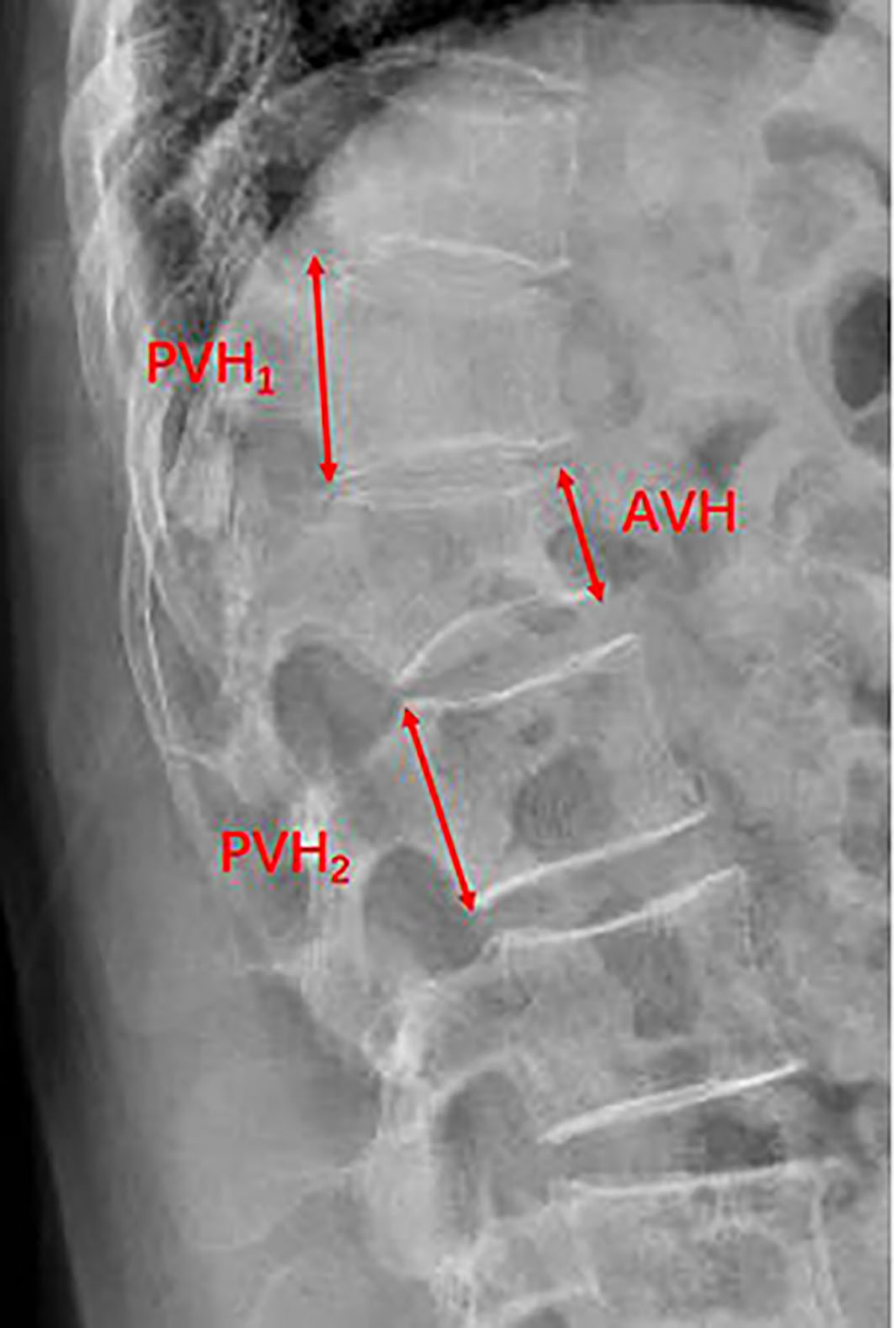
Plain radiograph showed the measurement of the anterior vertebral height (AVH) of the new fractured vertebral body, the posterior vertebral height (PVH_1_) of the adjacent cranial vertebral body, and the posterior vertebral height (PVH_2_) of the adjacent caudal vertebral body.

**Figure 3:**
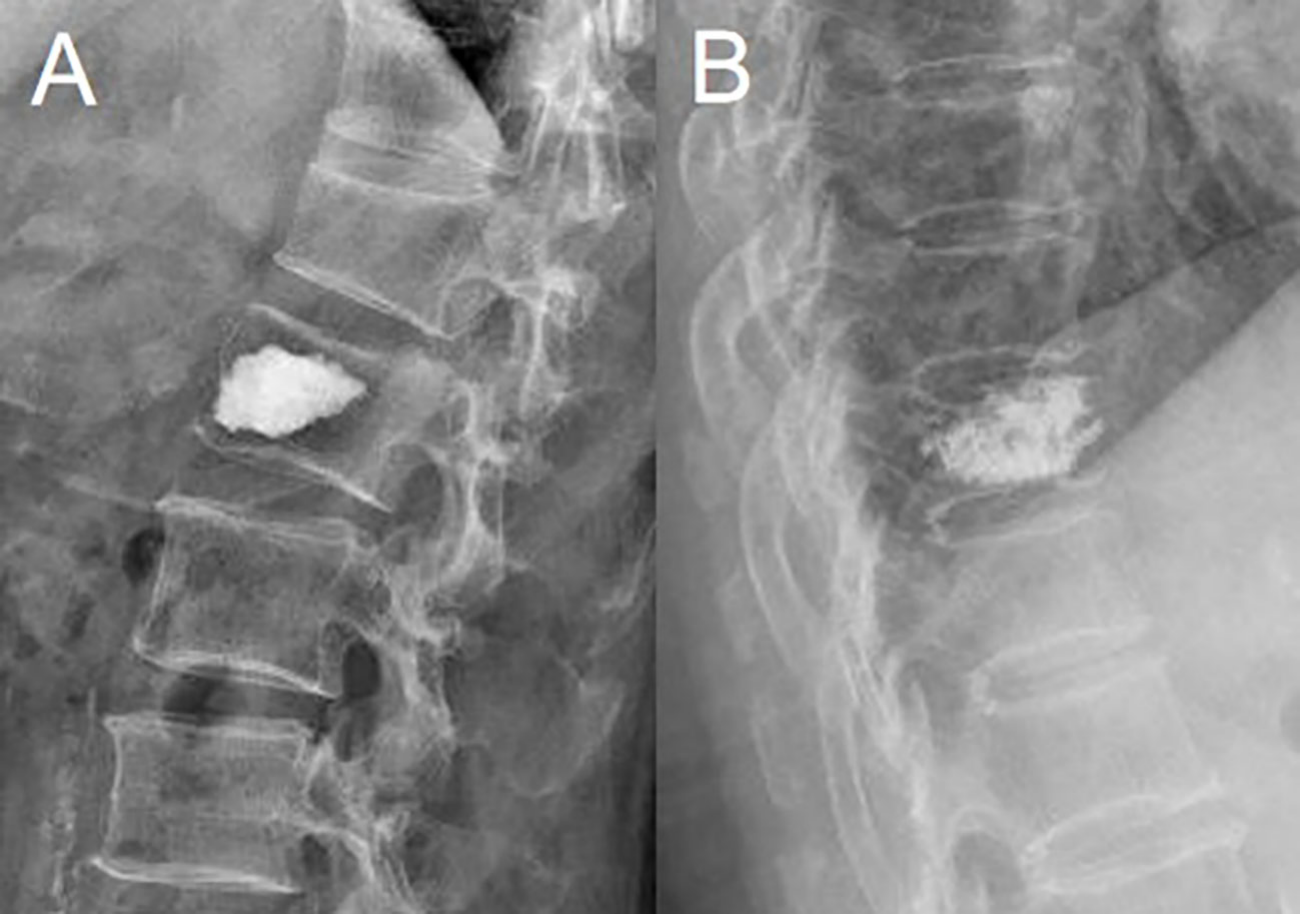
Cement distribution. **(A)** Compact type. **(B)** Trabecular type.

Age, gender, BMI, smoking, drinking, hypertension, diabetes, fracture location, surgical method, Hounsfield unit (HU) value, preoperative degree of anterior vertebral compression (DAVC), use of bisphosphonates, bone cement volume, bone cement leakage, and cement distribution were collected. Fracture location was divided into thoracolumbar (TL) junction and non-TL junction. Surgical method included PVP and PKP. HU value was an indicator of BMD and was used to diagnose osteoporosis (17). HU value was measured by an elliptical region of interest on the axial CT images at the first lumbar vertebral body. The HU value of L1 vertebral body was equal to the average value of the three axial slices: superior to the inferior endplate, the middle of the vertebral body, and inferior to the superior endplate (18) (**Figure 1**). If the first lumbar vertebral body fractured, the average HU value of the 12th thoracic vertebra and the second lumbar vertebral body was calculated. DAVC was an indicator of the degree of vertebral compression fracture. The measurement method of DAVC was the same as the previous study (19). The anterior vertebral height (AVH) of the new fractured vertebral body, the posterior vertebral height (PVH_1_) of the adjacent cranial vertebral body, and the posterior vertebral height (PVH_2_) of the adjacent caudal vertebral body were measured on lateral plain radiograph (**Figure 2**). DAVC of the fractured vertebral body was the ratio of the AVH to the mean of PVH_1_ and PVH_2_, with the formula DAVC = AVH/[(PVH_1_ + PVH_2_)/2] × 100%. DAVC was usually expressed as the A–P ratio. Cement distribution contained the compact type and the trabecular type (20) (**Figure 3**). In terms of the use of bisphosphonates, patients were given a dose of 5 mg of zoledronic acid, which was a type of bisphosphonate drug administered *via* an intravenous drip for at least 15 min. All the data obtained from the images were measured by two professional doctors.

### Statistical assessments

The data were processed by the SPSS 24 (IBM Corporation, Armonk, New York, USA). Risk factors of new VCF after PVP or PKP were obtained by univariate analysis and multivariate regression analysis. In the univariate analysis, continuous variables were presented as the mean with standard deviation or median with interquartile range, and categorical variables were expressed as the number with percentages. Continuous variables with normal distribution were compared by Student’s *t*-test; otherwise, the Mann–Whitney *U* test was used. Comparisons of categorical variables between the control and new VCF groups were conducted by the Chi-square or Fisher’s exact test. All variables with *p* < 0.05 on the basis of the results of univariable analysis were analyzed by multivariate regression analysis. *p* < 0.05 was considered to be statistically significant.

## Results

A total of 247 patients (203 women and 44 men) were included in the study. The mean age, BMI, and HU value of 247 patients were 69.58 ± 8.40 years, 22.92 ± 3.37 kg/m^2^, and 73.41 ± 30.30, respectively. Of 247 patients, 206 (83.4%) fractured at the TL junction. Of 247 patients, 67 (27.1%) underwent PKP whereas 180 (72.9%) underwent PVP (**Table 1**).

**Table 1:** Information of the study subjects and results of univariable analysis of the control and new VCF groups.

Variable	All patients (*n* = 247)	Control group (*n* = 224)	New VCF group (*n* = 23)	*p*
Age, years	69.58 ± 8.40	68.87 ± 8.27	76.48 ± 6.27	0.000*
Male, *n* (%)	44 (17.8)	38 (17.0)	6 (26.1)	0.422
BMI, kg/m^2^	22.92 ± 3.37	23.13 ± 3.33	20.84 ± 3.16	0.002*
Smoking, *n* (%)	33 (13.4)	28 (12.5)	5(21.7)	0.358
Drinking, *n* (%)	15 (6.1)	14 (6.3)	1 (4.3)	1.000
Hypertension, *n* (%)	98 (39.7)	91 (40.6)	7 (30.4)	0.341
Diabetes, *n* (%)	34 (13.8)	33 (14.7)	1 (4.3)	0.290
Fracture location, *n* (%)				0.030*
Non-TL junction	41 (16.6)	33 (14.7)	8 (34.8)	
TL junction	206 (83.4)	191 (85.3)	15 (65.2)	
Surgical method, *n* (%)				0.542
PVP	180 (72.9)	162 (72.3)	18 (78.3)	
PKP	67 (27.1)	62 (27.7)	5 (21.7)	
HU value	73.41 ± 30.30	76.98 ± 28.49	38.64 ± 25.36	0.000*
A–P ratio	0.70 ± 0.14	0.70 ± 0.14	0.75 ± 0.17	0.086
Bisphosphonates, *n* (%)	89 (36.0)	83 (37.1)	6 (26.1)	0.297
Cement volume, ml	4.47 ± 0.94	4.47 ± 0.95	4.50 ± 0.74	0.876
Cement leakage, *n* (%)	94 (38.1)	85 (37.9)	9 (39.1)	0.911
Cement distribution, *n* (%)				0.266
Compact type	102 (41.3)	90 (40.2)	12 (52.2)	
Trabecular type	145 (58.7)	134 (59.8)	11 (47.8)	

Values are expressed as the mean ± SD, number (%), or as otherwise indicated. BMI, body mass index; TL, thoracolumbar; PVP, percutaneous vertebroplasty; PKP, percutaneous kyphoplasty; HU, Hounsfield unit; A–P, anterior–posterior; VCF, vertebral compression fracture.

* p < 0.05 versus the control group.

Twenty-three patients (23/247, 9.3%) had new VCF after PVP or PKP during the follow-up period. Of the 23 patients, 6 patients (26.1%) were male and 17 patients (73.9%) were female. The mean age, BMI, and HU value of the 23 patients were 76.48 ± 6.27 years, 20.84 ± 3.16 kg/m^2^, and 38.64 ± 25.36, respectively. Of the new VCF, 65.2% (15/23) and 34.8% (8/23) occurred in the TL junction and the non-TL junction, respectively. Nine patients (39.1%) developed cement leakage (**Table 1**).

In the univariate analysis, no significant difference was found in gender (*p* = 0.422), smoking (*p* = 0.358), drinking (*p* = 1.000), hypertension (*p* = 0.341), and diabetes (*p* = 0.290) between the control and new VCF groups except age (*p* < 0.001) and BMI (*p* = 0.002). There was no significant difference between the two groups in terms of surgical method (*p* = 0.542), A–P ratio (*p* = 0.086), bisphosphonates (*p* = 0.297), bone cement volume (*p* = 0.876), bone cement leakage (*p* = 0.911), and cement distribution (*p* = 0.266) except fracture location (*p* = 0.030) and HU value (*p* < 0.001) (**Table 1**). Multivariate regression analysis found that age (OR = 1.061; 95% CI, 0.992–1.135, *p* = 0.082), BMI (OR = 0.917; 95% CI, 0.794–1.058, *p* = 0.234), and fracture location (OR = 0.598; 95% CI, 0.192–1.858, *p* = 0.374) were not significantly associated with new VCF after PVP or PKP. Only HU value was a negative independent factor for new VCF (OR = 0.963; 95% CI, 0.943–0.984, *p* = 0.001) and significantly weakened the odds risk of new VCF (**Table 2**).

**Table 2:** Multivariable regression analysis for the risk of new VCF.

Variable	B	SE	OR (95% CI)	*p*
Age, years	0.060	0.034	1.061 (0.992–1.135)	0.082
BMI, kg/m^2^	−0.087	0.073	0.917 (0.794–1.058)	0.234
Fracture level	−0.514	0.578	0.598 (0.192–1.858)	0.374
HU value	−0.037	0.011	0.963 (0.943–0.984)	0.001

BMI, body mass index; HU, Hounsfield unit; VCF, vertebral compression fracture; B, unstandardized B coefficient; SE, standard error; OR, odds ratio; CI, confidence interval.

## Discussion

New VCF is a severe adverse event in patients who underwent PVP or PKP for OVCF. Some studies reported that the incidence of new VCF after PVP or PKP was 11.5%–34.8% (19, 21–23). In our study, the incidence of new VCF was 9.3% (23/247), which was lower than the above data. Compared with other studies, perhaps this study only included the initial single-segment VCF, which reduced the incidence of new VCF. Cao et al. (24) concluded that multiple segment VCF increased the incidence of the new VCFs after vertebroplasty. Previous studies showed several risk factors of new vertebral compression fracture after PVP or PKP. Ning et al. (25) reported that risk factors of new VCF after PKP for OVCF were gender, zoledronic acid, and previous fracture history rather than BMI, smoking, alcohol, hypertension, and diabetes. Bian et al. (26) concluded that age, HU value, TL junction fracture, and cement leakage were correlated with new VCF after PVP in patients with OVCF. In this study, only a low HU value was the independent risk factor of new VCF after PVP or PKP by multivariate regression analysis.

Osteoporosis usually occurs in the elderly. Age is considered to be associated with osteoporosis. However, whether age is a risk factor for new VCF remains controversial. Zhang et al. (27) reported that age was correlated with new VCF after PVP. However, a study conducted by Li et al. (23) demonstrated that age was not associated with new OVCF after vertebroplasty. In our study, a significant difference was found in age between the control and new VCF groups. However, age was not associated with new VCF after PVP or PKP by multivariate regression analysis.

Whether BMI is associated with new VCF is inconclusive. Ahn et al. (28) suggested that BMI was a negative independent risk factor for new VCF after PVP. However, Tanaka et al. (29) indicated that overweight and underweight were associated with fractures. In the study of 132 patients, Lee et al. (21) concluded that BMI was not related to the new VCF after PVP or PKP for OVCF. Mao et al. (12) found that BMI was not a risk factor of secondary fracture after PVP for OVCF. Moreover, in the meta-analysis, Zhai et al. (30) reported that BMI was not correlated with subsequent fracture for OVCF after PVP. In our study, there was a significant difference in BMI between the control and new VCF groups. However, multivariate regression analysis showed that BMI was not correlated with new VCF after PVP or PKP.

Spinal fractures usually occur in the TL junction. Our study showed that 83.4% of 247 patients had fractures in the TL junction. However, whether TL junction is a risk factor for new VCF is still uncertain. A research conducted by Bian et al. (31) indicated that TL junction was strongly associated with new VCF after PKP. Zhang et al. (32) retrospectively reviewed 421 patients in order to identify the risk factors for new VCF, and concluded that the TL junction was not an independent risk factor for new VCF, which was in line with our study.

BMD was used as an indicator of osteoporosis. Previous studies showed that a low *T*-score was correlated with new VCF after PVP (33, 34). However, Yang et al. (35) indicated that low BMD was not associated with refracture. Moreover, *T*-score measured by dual-energy x-ray absorptiometry (DXA) may be high due to spinal degeneration or deformity (36). Schreiber et al. (18) reported that the HU value measured by CT could be used to assess BMD. Ji et al. (37) indicated that HU value significantly associated with refracture after OVCF. In our study, we concluded that HU value was a negative independent factor for new VCF, in line with a previous study (31). Therefore, patients with a low HU value should receive appropriate antiosteoporosis treatment so as to increase BMD, which could reduce the risk of new VCF after PVP or PKP for OVCF.

There were some limitations in our study. Firstly, to some extent, there may be selection bias in this retrospective study. Secondly, the sample size was small in this study, with only 23 patients with new VCF after PVP or PKP. Prospective multicenter randomized controlled trials with a large sample size are still needed to better analyze the risk factors of new VCF after PVP or PKP in the future.

## Conclusion

A low HU value was an independent risk factor of new VCF after PVP or PKP. Patients with a low HU value are likely to have new VCF after PVP or PKP for OVCF.

## Data availability statement

The raw data supporting the conclusions of this article will be made available by the authors, without undue reservation.

## Ethics statement

This study was reviewed and approved by the Ethics Committee of China-Japan Union Hospital of Jilin University. The patients/participants provided their written informed consent to participate in this study.

## Author contributions

YC and HW designed the study. YC collected the data and wrote the manuscript. XC designed the figures and provided valuable comments. YC and HW revised the manuscript. The final manuscript was approved by all authors. All authors contributed to the article and approved the submitted version.

## Conflict of interest

The authors declare that the research was conducted in the absence of any commercial or financial relationships that could be construed as a potential conflict of interest.

## Publisher’s note

All claims expressed in this article are solely those of the authors and do not necessarily represent those of their affiliated organizations, or those of the publisher, the editors and the reviewers. Any product that may be evaluated in this article, or claim that may be made by its manufacturer, is not guaranteed or endorsed by the publisher.
